# Variation in Plasma Levels of TRAF2 Protein During Development of Squamous Cell Carcinoma of the Oral Tongue

**DOI:** 10.3389/fonc.2021.753699

**Published:** 2021-11-23

**Authors:** Xiaolian Gu, Philip Coates, Lixiao Wang, Baris Erdogan, Amir Salehi, Nicola Sgaramella, Katarina Zborayova, Karin Nylander

**Affiliations:** ^1^ Department of Medical Biosciences, Umeå University, Umeå, Sweden; ^2^ Research Centre for Applied Molecular Oncology, Masaryk Memorial Cancer Institute, Brno, Czechia; ^3^ Department of Clinical Sciences, Umeå University, Umeå, Sweden

**Keywords:** TRAF2, plasma protein, tongue cancer, prediction, biomarker

## Abstract

As early detection is crucial for improvement of cancer prognosis, we searched for biomarkers in plasma from individuals who later developed squamous cell carcinoma of the oral tongue (SCCOT) as well as in patients with an already established SCCOT. Levels of 261 proteins related to inflammation and/or tumor processes were measured using the proximity extension assay (PEA) in 179 plasma samples (42 collected before diagnosis of SCCOT with 81 matched controls; 28 collected at diagnosis of SCCOT with 28 matched controls). Statistical modeling tools principal component analysis (PCA) and orthogonal partial least square - discriminant analysis (OPLS-DA) were applied to provide insights into separations between groups. PCA models failed to achieve group separation of SCCOT patients from controls based on protein levels in samples taken prior to diagnosis or at the time of diagnosis. For pre-diagnostic samples and their controls, no significant OPLS-DA model was identified. Potentials for separating pre-diagnostic samples collected up to five years before diagnosis (*n* = 15) from matched controls (*n* = 28) were seen in four proteins. For diagnostic samples and controls, the OPLS-DA model indicated that 21 proteins were important for group separation. TNF receptor associated factor 2 (TRAF2), decreased in pre-diagnostic plasma (< 5 years) but increased at diagnosis, was the only protein showing altered levels before and at diagnosis of SCCOT (*p*-value < 0.05). Taken together, changes in plasma protein profiles at diagnosis were evident, but not reliably detectable in pre-diagnostic samples taken before clinical signs of tumor development. Variation in protein levels during cancer development poses a challenge for the identification of biomarkers that could predict SCCOT development.

## Introduction

Proteins found in blood plasma can be informative of health status and disease risk (1). Proteins can enter plasma through purposeful secretion or leakage from damaged and dead cells (1). In the search for cancer biomarkers, plasma proteins have long been considered an attractive resource (2, 3). A number of FDA (US Food and Drug Administration)-approved plasma protein biomarkers for cancer are currently used in clinical practice, such as prostate-specific antigen (PSA) for prostate cancer, alpha-fetoprotein (AFP) for testicular cancer, cancer antigen 125 (CA-125) for ovarian cancer, CA 15-3 for breast cancer and CA 19-9 for pancreatic cancer (3, 4). Despite their approved application, insufficient specificity for cancer diagnosis or management is well recognized (2, 3).

Oral cancer is a common disease worldwide, representing approximately 2% of all new global cancer cases in 2018 (5). According to The Swedish Head and Neck Cancer Register (SweHNCR), the number of cases increased slightly during the years 2008 to 2017, with about 400 new cases each year in Sweden (6). Squamous cell carcinoma of the oral tongue (SCCOT) is the main sub-group of oral cancer. For this sub-group of patients, the relative five-year survival is 88% for patients with stage I tumors, 73% for stage II, 46% for stage III and 33% for stage IV [data from SweHNCR (6)]. Cancer-specific changes that can be detected at an early phase of cancer development are of great value for increasing the chances of successful treatment and better prognosis (7).

In a previous study, we found that plasma proteins are promising diagnostic markers for SCCOT, more promising than circulating miRNAs for this purpose (8), but no clinically approved biomarker exists as yet for oral cancer (4, 9). Based on comparison of blood samples taken from patients with oral SCC at diagnosis with samples from healthy controls Liu et al. reported that plasma-derived inflammatory proteins could be useful, but no pre-diagnostic samples were investigated to assess the potential of the proteins in early diagnosis (10). In our recent study that focused on interleukin 1 receptor antagonist (IL-1Ra), a commonly suggested cancer biomarker, we found that IL-1Ra levels in pre-diagnostic samples has low potential as a predictive biomarker for SCCHN (11). As protein profiles prior to diagnosis remain relatively unexplored it is unclear whether markers of an existing carcinoma are altered prior to development of the visible tumor.

To address this issue, we analyzed a total of 261 proteins known to be involved in inflammation and/or tumor processes in plasma samples taken before and at diagnosis of SCCOT. The pre-diagnostic samples available from patients who subsequently developed clinical SCCOT had been collected from 6 months up to 22 years before diagnosis. By investigating if any proteins had altered levels before diagnosis, and comparing patterns of changes between pre-diagnostic and diagnostic plasma samples we searched for predictive biomarkers.

## Materials and Methods

### Pre-Diagnostic SCCOT Samples and Matched Controls

**Figure 1:**
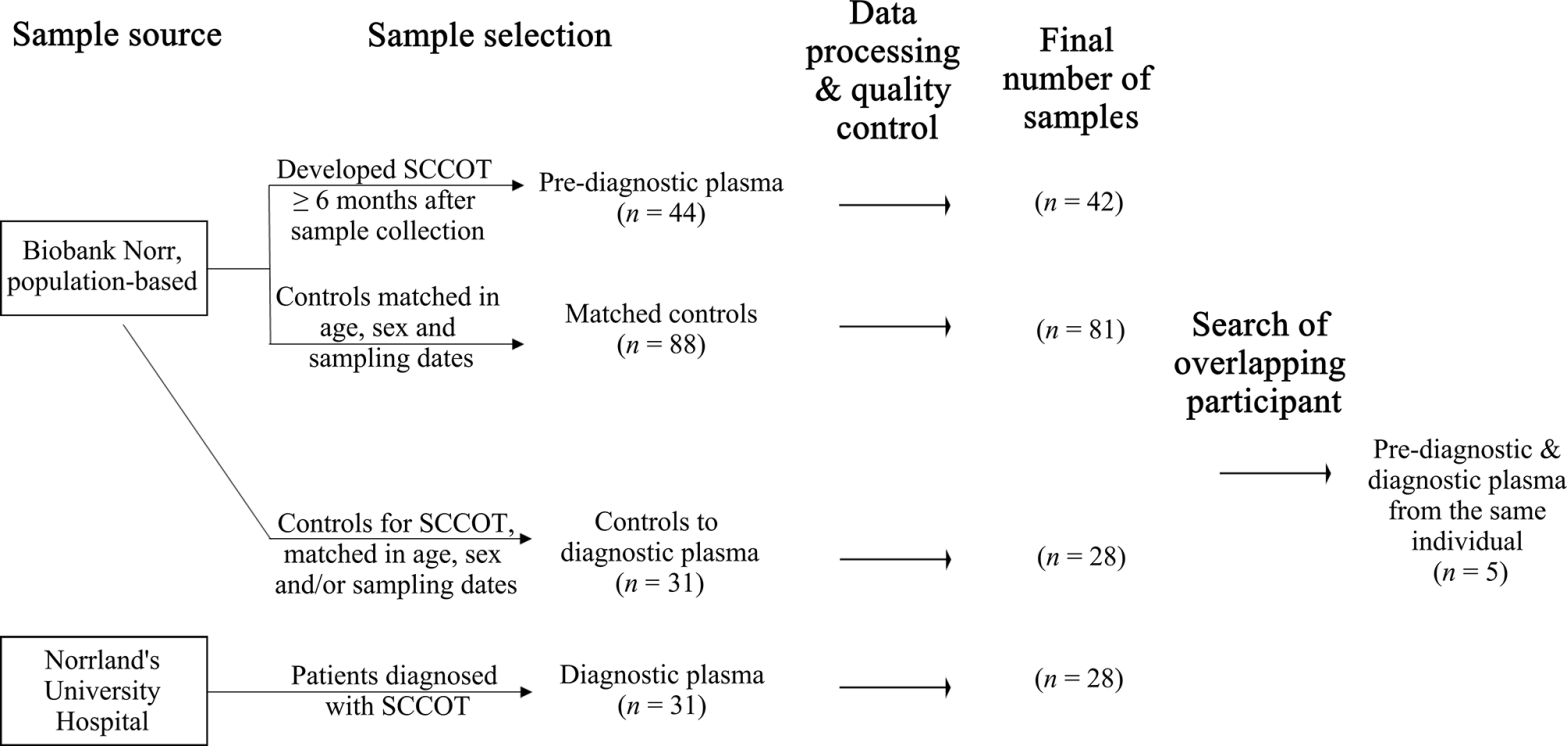
Sample selection and the final sample set with qualified data.

Plasma samples from the Västerbotten Intervention Programme (VIP) and the Northern Sweden Monica Project (MONICA) collected in Biobank Norr, Umeå, were used. These are large ongoing population-based cohorts established in the late 1980’s (12). As summarized in **Figure 1**, 44 plasma samples from subjects that developed SCCOT six months or longer after sample collection were selected. The cases were matched with two age- (± 12 months), sex- and sampling dates- (± 12 months) matched healthy controls that had not developed any kind of cancer, in total 88 controls. After at least 8 hours of fasting, according to a standardized protocol, all samples were collected in EDTA tubes in the morning. Samples were aliquoted and frozen within 1 h of collection, either directly at −80°C or first at −20°C for up to one week before being transferred to a central storage facility.

### SCCOT Samples and Controls

After informed consent, plasma from 31 primary SCCOT patients was collected in EDTA tubes at Norrland’s University Hospital in Umeå, Sweden. Pre-diagnostic plasma samples were available for five of these patients (**Figure 1**). Samples were collected before start of treatment. All but 8 patients were fasting at least 8 hours before sample collection. Plasma was aliquoted and stored frozen at −80°C until further analysis. Age- (± 5 years), sex- and/or sampling date- (± 5 years) matched healthy controls were chosen from Biobank Norr. Ethical permission for the study had been granted (Dnr 08-003M) and the project was performed according to the principles of the Declaration of Helsinki.

### Plasma Sample Analysis and Data Processing

Plasma samples were analyzed with three different Olink Multiplex panels (Olink Proteomics, Uppsala, Sweden), each containing 92 proteins representing cell regulation, immune response and immune oncology. With 15 proteins being present on two of the panels, the total number of proteins investigated was 261. A list of the assays can be seen in **Table S1**. All samples were sent to the Clinical biomarker facility, Science for Life Laboratory (Uppsala, Sweden) for analysis. The technique for the multiplex system is proximity extension assay (PEA), which is based on pairs of antibodies linked to oligonucleotides that are brought in proximity when they bind to their target protein. The oligonucleotide is extended by DNA polymerase and analyzed with qPCR. The copy numbers formed are proportional to the concentration of the antigen in the sample. Results are presented as normalized protein expression (NPX), which is an arbitrary unit on a log2 scale. Internal and external controls were included in each run. Information about quality control of the data can be found online: https://www.olink.com/faq/how-is-quality-control-of-the-data-performed/. The limit of detection (LOD) is calculated separately for each Olink assay and sample plate. Values below LOD were classified as LOD.

For the dataset comprising pre-diagnostic samples and matched controls, two pre-diagnostic samples did not pass quality control and were thus excluded, together with their matched controls. Three controls for pre-diagnostic samples were also excluded due to low quality. For the dataset comprising diagnostic samples and the corresponding controls, three diagnostic and three controls did not pass quality control and were excluded, resulting in 18 pairs of age-, sex- and sampling date-matched samples and 10 pairs of partially matched samples (**Table S2**). Finally, plasma protein data were available from 42 pre-diagnostic cases with 81 matched controls, and from 28 patients at diagnosis with 28 partially matched controls. Clinical information for patients is shown in **Table 1**.

**Table 1:** Clinical information for patients with pre-diagnostic or diagnostic plasma samples.

ID	Group	Months before diagnosis	Age	Gender	TNM	BMI group	Fasting state	Smoking	Alcohol
1	<5 years before diagnosis	6	60	Male		NA	Yes	NA	NA
2		7	47	Male		Overweight	Yes	NS/P/O	NA
3		10	59	Male		Overweight	Yes	FS	Alcohol
4		17	60	Male		Obese	Yes	FS	Alcohol
5		19	60	Female		Overweight	Yes	NS/P/O	NA
6		25	60	Male		Overweight	Yes	S	NA
7		30	40	Female		Normal	Yes	FS	Alcohol
8		35	61	Male		Overweight	Yes	NS/P/O	NA
9		37	60	Female		Obese	Yes	NS/P/O	NA
10		37	51	Male		Overweight	Yes	FS	NA
11		40	60	Female		Normal	Yes	NS/P/O	Alcohol
12		47	50	Female		Obese	Yes	FS	Alcohol
13		51	60	Male		Overweight	Yes	FS	Alcohol
14		52	60	Female		Normal	Yes	NS/P/O	Alcohol
15		58	50	Female		Normal	Yes	S	NA
16	5-15 years before diagnosis	61	60	Male		Overweight	Yes	S	NA
17		62	40	Male		Overweight	Yes	NS/P/O	Alcohol
18		68	68	Female		Normal	Yes	NA	NA
19		82	65	Female		Normal	Yes	NS/P/O	NA
20		88	50	Male		Overweight	Yes	S	NA
21		111	60	Male		Normal	Yes	NS/P/O	Alcohol
22		114	60	Female		Obese	Yes	FS	Alcohol
23		125	60	Female		Obese	Yes	S	NA
24		126	40	Female		Overweight	Yes	S	NA
25		136	60	Male		Obese	Yes	NS/P/O	NA
26		151	55	Female		Obese	Yes	NA	NA
27		155	66	Female		Overweight	Yes	NA	NA
28		168	60	Male		Overweight	Yes	NS/P/O	NA
29		169	60	Female		Overweight	Yes	NS/P/O	NA
30		174	50	Male		Normal	Yes	FS	NA
31		176	60	Male		Overweight	Yes	NS/P/O	NA
32	>15 years before diagnosis	189	63	Male		Extremely Obese	Yes	S	NA
33		194	63	Female		Normal	Yes	NA	NA
34		199	68	Male		Overweight	Yes	S	NA
35		204	57	Female		NA	Yes	NA	NA
36		215	58	Female		Obese	Yes	NA	NA
37		223	50	Male		Normal	Yes	S	NA
38		230	60	Male		Overweight	Yes	FS	NA
39		232	50	Male		Overweight	Yes	S	NA
40		233	50	Male		Overweight	Yes	S	NA
41		263	50	Female		Normal	Yes	NS/P/O	NA
42		270	40	Male		Obese	Yes	NS/P/O	NA
43	At diagnosis	0	69	Male	T4aN0M0	Normal	NA	S	Alcohol
44		0	62	Male	T2N0M0	Overweight	NA	FS	NA
45		0	19	Female	T4N0M0	Overweight	NA	FS	NA
46		0	64	Female	T1N0M0	Normal	NA	NA	NA
47		0	64	Male	T4aN2cM0	Normal	NA	S	Alcohol
48		0	31	Female	T1N0M0	Overweight	Yes	S	Alcohol
49		0	66	Male	T2N0M0	Normal	Yes	NS/P/O	No alcohol
50		0	54	Male	T4aN2bM0	Normal	Yes	S	Alcohol
51		0	74	Female	T2N0M0	Overweight	Yes	NS/P/O	No alcohol
52		0	71	Female	T2N0M0	Normal	Yes	NS/P/O	Alcohol
53		0	50	Male	T2N1M0	Normal	Yes	S	Alcohol
54		0	80	Male	T1N0M0	Normal	Yes	NS/P/O	Alcohol
55		0	69	Female	T1N0M0	Obese	Yes	FS	Alcohol
56		0	42	Female	T1N1M0	Normal	Yes	NS/P/O	Alcohol
57		0	84	Female	T2N0M0	Normal	Yes	FS	Alcohol
58		0	65	Male	T1N0M0	Normal	Yes	S	Alcohol
59		0	73	Female	T1N0M0	Extremely Obese	NA	FS	No alcohol
60		0	73	Female	T1N0M0	Overweight	Yes	NS/P/O	Alcohol
61		0	71	Female	T1N0M0	Overweight	Yes	NS/P/O	Alcohol
62		0	52	Male	T4aN2bM0	Extremely Obese	NA	NS/P/O	Alcohol
63		0	72	Female	T2N0M0	Normal	Yes	S	Alcohol
64		0	71	Female	pT4aN2bM0	Overweight	Yes	S	Alcohol
65		0	62	Male	pT2pN2bM0	Obese	Yes	S	No alcohol
66		0	71	Male	T4aN0M0	Normal	Yes	FS	Alcohol
67		0	54	Female	T1N0M0	Extremely Obese	NA	NS/P/O	No alcohol
68		0	36	Male	T2N0M0	Extremely Obese	Yes	NS/P/O	No alcohol
69		0	39	Male	T2N0M0	Extremely Obese	Yes	S	Alcohol
70		0	69	Male	T2N0M0	Normal	Yes	S	Alcohol

NA, not available; NS/P/O, nonsmoker, party smoker or occasional smoker; FS, former smoker; S, smoker.

### Data Analysis

For multivariate data analysis we used Simca 16 (MKS Data analytics Solutions, Umea, Sweden) (13). Unsupervised principal component analysis (PCA) was performed to overview sample distributions based on three panels of protein levels. Orthogonal partial least square - discriminant analysis (OPLS-DA) was conducted to identify proteins that were important for group differences (cases vs. controls). For each OPLS-DA model, analysis of variance of cross-validated predictive residuals (CV-ANOVA) was performed to assess model reliability. A model with CV-ANOVA *p*-value < 0.05 was considered significant. Proteins with variable influence of projection (VIP) value higher than 1, as well as absolute correlation coefficient between the model and original data |p(corr)| > 0.4 were regarded important for group discrimination (13).

Univariate statistical methods were applied to compare levels of the top discriminatory proteins between groups in order to visualize the changes in single factors. The analysis was conducted in IBM SPSS Statistics 25 (IBM Corp., Armonk, NY, USA). For comparison between two groups of continuous variables, Mann-Whitney U test for non-paired samples were used, as controls were not perfectly matched. A two-sided *p*-value < 0.05 was considered significant. Distribution of individual data was shown in bean plots generated by BoxplotR (14). Discrimination ability of single potential protein biomarkers was assessed *via* receiver operating characteristic (ROC) curve and area under the curve (AUC) value. Sensitivity and specificity at cutoff levels were also calculated.

## Results

### Protein Profiles in Pre-Diagnostic and Diagnostic SCCOT

**Figure 2:**
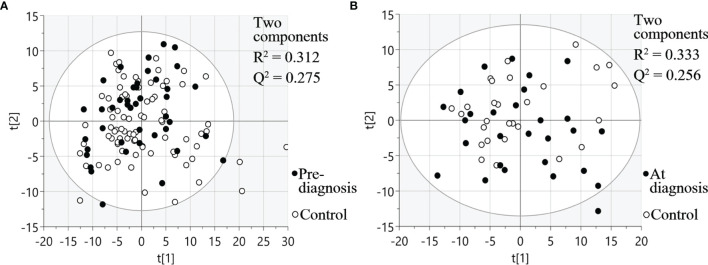
PCA plots visualizing protein expression profiles in different samples. **(A)** Pre-diagnostic plasma samples and matched controls. **(B)** Diagnostic plasma and controls. t[1] and t[2] are scores of the first two principal components.

Three different multiplex panels consisting of 92 different proteins each were used for analysis of plasma taken between six months up to 22 years before SCCOT diagnosis (pre-diagnostic plasma), plasma collected at diagnosis from patients with SCCOT (diagnostic plasma) and from matched controls without any history of cancer. PCA, an unsupervised multivariate statistical analysis method, showed that pre-diagnostic samples exhibited similar protein profiles to their age- and sex-matched controls (**Figure 2A**), and protein profiles were similar also between diagnostic plasma and their controls (**Figure 2B**). Pre-diagnostic samples were further divided into three subgroups according to the interval from collection date to cancer diagnosis date: < 5 years before diagnosis, 5 to 15 years before diagnosis and > 15 years before diagnosis, showing no separation among the three subgroups based on time to tumor detection (data not shown).

For diagnostic samples, the associations between clinicopathological features and protein profiles were also evaluated by PCA plots (**Figure S1**). No obvious sample groupings according to TNM stage, T stage, node status or alcohol consumption were found.

### Identification of Discriminant Factors Using OPLS-DA

**Figure 3:**
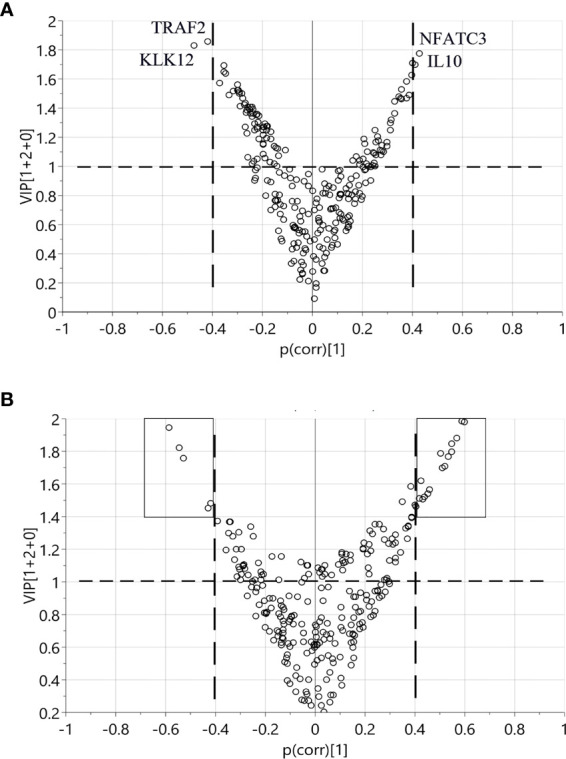
Identification of discriminant factors between two groups of samples according to OPLS-DA. Variables with |*p(corr)*| > 0.4 and *VIP* > 1 were selected. **(A)** Comparison between < 5 years pre-diagnostic samples and the matched controls. Four proteins selected as potential discriminant factors were shown. **(B)** Comparison between plasma at diagnosis and the matched controls. The black boxes indicate the 21 proteins as discriminating variables.

Using OPLS-DA modelling, we tried to identify proteins that could contribute to group separation. For pre-diagnostic samples and controls, no significant OPLS-DA models were identified (CV-ANOVA *p*-value = 1). Nevertheless, we considered proteins with |p(corr)| > 0.4 and VIP > 1 as potential discriminant factors. As shown in **Table 2**, when we compared all pre-diagnostic samples to their matched controls, IL8 was the only protein that showed potential for group separation. When comparing samples collected < 15 years before diagnosis to their matched controls, two potential discriminant proteins were identified (IL8 and MCP-3). For samples collected < 5 years before diagnosis, a different set of potential discriminant proteins was identified (NFATC3, IL10, TRAF2 and KLK12) (**Figure 3A** and **Table 2**). Next, we compared diagnostic plasma to the corresponding controls. The OPLS-DA model (three components, R^2^ = 0.864, Q^2^ = 0.613, CV-ANOVA *p*-value < 0.001) indicated that 21 proteins (measured by 22 assays) were important for group separation (**Figure 3B** and **Table 2**).

**Table 2:** List of discriminant factors identified from comparisons between different groups of samples.

Comparison with controls		Discriminant factor^#^	Olink ID	p(corr)	VIP
**Pre-diagnostic plasma**	0.5 to 22 years	IL8?	OID00752	0.405565	1.95369
	<15 years	IL8?	OID00752	0.427393	2.19945
		MCP-3?	OID00755	0.440704	2.15198
	<5 years	NFATC3?	OID00973	0.42625	1.77496
		IL10?	OID00809	0.409816	1.69605
		TRAF2?	OID00963	-0.418688	1.85981
		KLK12?	OID01395	-0.472765	1.83215
**Diagnostic plasma**		HGF	OID00803	0.599398	1.97931
		CPXM1	OID01378	0.588331	1.9842
		MMP7	OID00814	0.567931	1.88234
		ANGPT1	OID00760	0.548139	1.84904
		IL6	OID00947	0.547857	1.79925
		CLEC4D	OID00988	0.532688	1.76693
		IL6	OID00763	0.520751	1.71107
		NOS3	OID00777	0.511162	1.69755
		TNFSF14	OID00787	0.502889	1.78809
		TREM1	OID00991	0.4582	1.56557
		MCP-3	OID00755	0.452792	1.54159
		CXCL12	OID01008	0.43792	1.52199
		BCL2L11	OID01316	0.429986	1.50767
		TRAF2	OID00963	0.422922	1.6181
		ATG4A	OID01370	0.416763	1.51175
		LAP TGF-beta-1	OID00785	0.404186	1.46035
		PDGF subunit B	OID00790	0.401904	1.47148
		FASLG	OID00792	-0.41823	1.48066
		ICOSLG	OID00828	-0.429	1.45444
		TWEAK	OID00789	-0.52812	1.75852
		ITGA11	OID01021	-0.54414	1.82026
		LRRN1	OID01362	-0.58635	1.94583

^#^Discriminant factors selected from non-significant OPLS-DA models were marked with “?”.

### Longitudinal Trend of Change in Plasma Proteins

**Figure 4:**
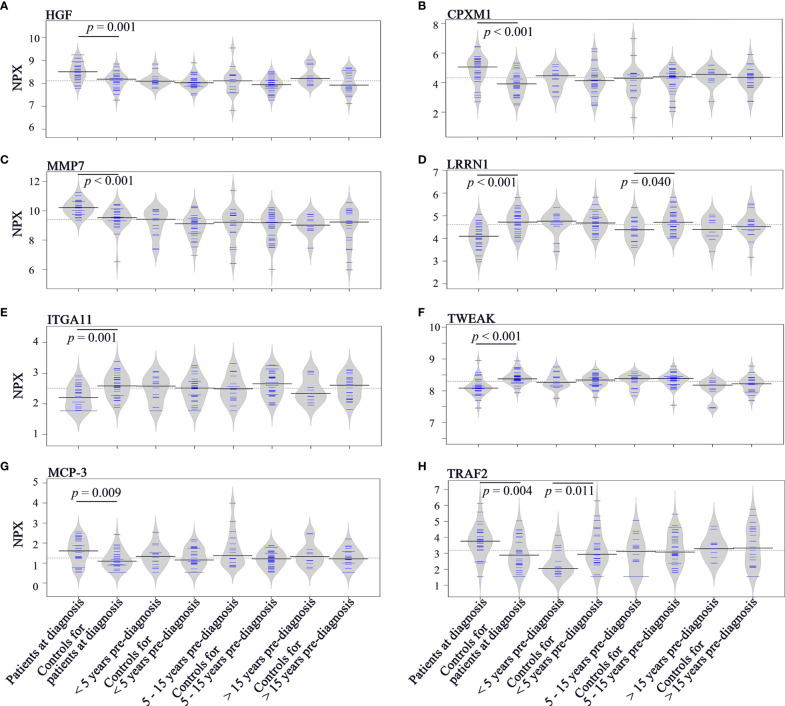
Bean plots showing protein levels in different groups of plasma samples. **(A)** HGF. **(B)** CPXM1. **(C)** MMP7. **(D)** LRRN1. **(E)** ITGA11. **(F)** TWEAK. **(G)** MCP-3. **(H)** TRAF2. The grey area shows the estimated density of distribution. The horizontal lines within the grey area show individual observations, while the black line is the median.

To investigate the longitudinal trend of change in protein levels long before diagnosis to diagnosis of cancer, levels of the top discriminatory proteins in diagnostic plasma, < 5 years before diagnosis, 5 - 15 years before diagnosis, > 15 years before diagnosis were compared to their respective controls. According to the p(corr) value (correlation coefficient between the model and the original data), HGF (Hepatocyte Growth Factor), CPXM1 (Carboxypeptidase X, M14 Family Member 1) and MMP7 (Matrix Metallopeptidase 7) are the top three positively correlated factors (**Table 2**) and each protein showed statistically different levels (*p* < 0.01) in diagnostic samples and controls (**Figures 4A–C**). However, no differences were detected in pre-diagnostic samples compared to their matched controls, regardless of interval to diagnosis (*p* > 0.05). The top three negatively correlated proteins were LRRN1 (Leucine Rich Repeat Neuronal 1), ITGA11 (Integrin Subunit Alpha 11) and TWEAK (TNF Superfamily Member 12) (**Table 2**) and (**Figures 4D–F**). Of these, LRRN1 was the only downregulated protein in a subgroup of pre-diagnostic samples (5-15 years before diagnosis) compared to the corresponding controls (**Figure 4D**).

Longitudinal trends of change in MCP-3 (C-C Motif Chemokine Ligand 7) and TRAF2 (TNF Receptor Associated Factor 2), the two proteins showing discriminatory potential in analysis of both diagnostic and pre-diagnostic plasma, were also investigated. As shown in **Figure 4G**, levels of MCP-3 were upregulated in diagnostic samples compared to matched controls (*p* = 0.009). However, no significant differences in MCP-3 levels were identified for the three subgroups of pre-diagnostic samples compared to their controls (*p* > 0.05). TRAF2 was downregulated in < 5 years pre-diagnostic samples (*p* = 0.011) but upregulated in diagnostic samples (*p* = 0.004) (**Figure 4H**).

ROC curve for < 5 years pre-diagnostic plasma and their controls revealed that TRAF2 had acceptable discrimination ability for the prediction of SCCOT (AUC = 0.736, *p* = 0.012). At cutoff value of 2.5 NPX, the specificity was 0.733 and the sensitivity was 0.714.

Performance of potential diagnostic markers are shown in **Table S3**. MMP7, TWEAK and LRRN1 showed good discrimination ability (0.8 ≤ AUC < 0.9), whereas other proteins only showed acceptable discrimination ability (0.7 ≤ AUC < 0.8).

### Samples From the Same Patient Before and at Diagnosis of SCCOT

**Figure 5:**
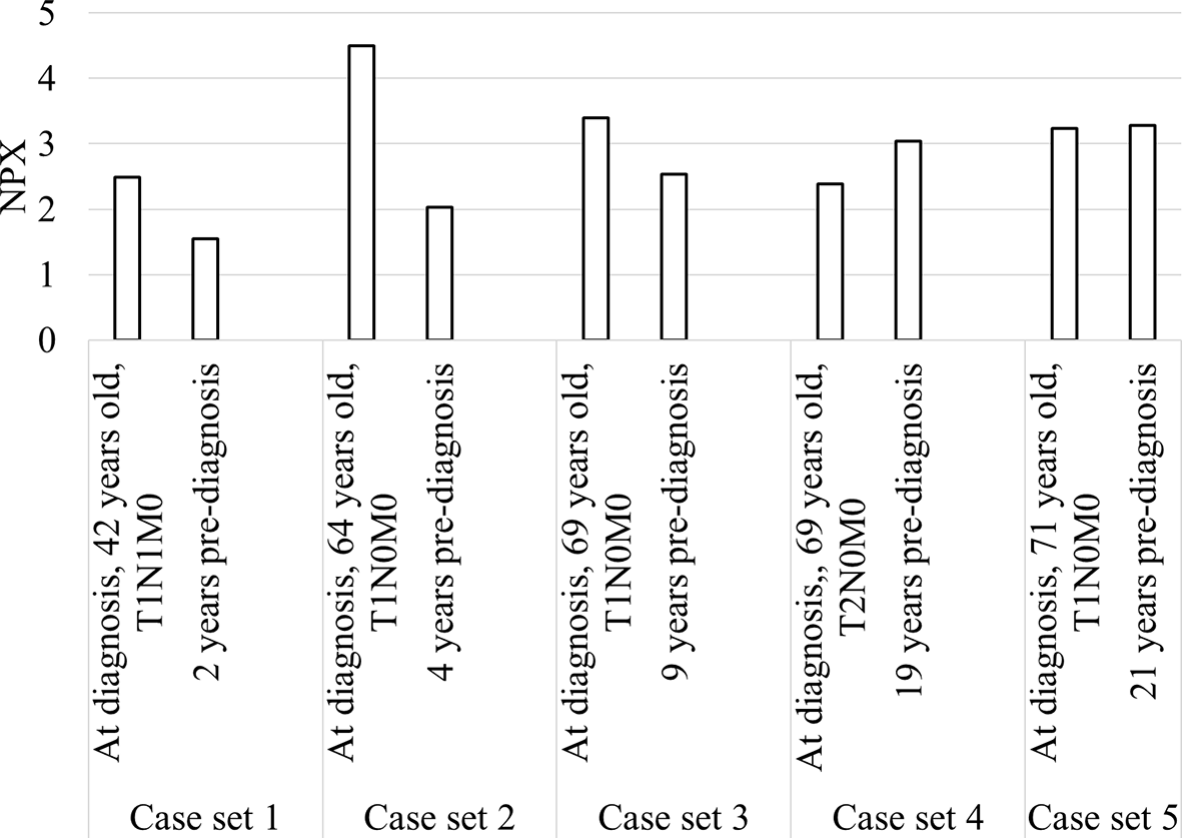
Bar chart showing TRAF2 levels in the five patients with diagnostic and pre-diagnostic plasma.

Longitudinal trends in changes in protein levels can be investigated by following the same patient over time, and a pre-diagnostic sample was available for five of our patients, ranging from 2 to 21 years before diagnosis (**Figure 5**). An increase in diagnostic plasma compared to pre-diagnostic plasma was seen for cases 1-3, with plasma samples taken 2, 4 and 9 years respectively before diagnosis. For the other two cases with pre-diagnostic samples collected 19 and 21 years before diagnosis, TRAF2 levels at diagnosis were slightly lower or similar to the pre-diagnostic samples.

### TRAF2 Levels and Clinical Features

**Figure 6:**
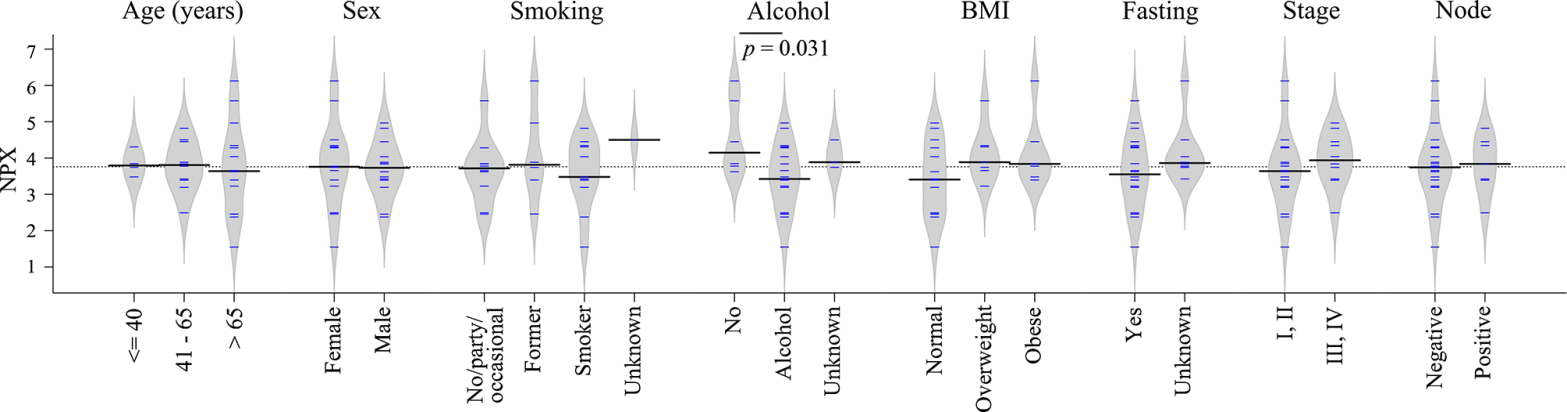
Bean plots showing TRAF2 levels in patients with different clinical features. The grey area shows the estimated density of distribution. The horizontal lines within the grey area show individual observations, while the black line is the median.

Clinical information such as age, sex, smoking, alcohol, body mass index (BMI), fasting state, tumor stage and node status was available for the majority of diagnostic samples, and the impact of these clinical factors on TRAF2 levels were studied. As shown in **Figure 6**, no significant difference in TRAF2 levels was demonstrated except for alcohol consumption (*p* = 0.031). Considering TRAF2 levels in the controls matched to diagnostic plasma, no significant difference regarding alcohol consumption was seen (**Figure S2**). Due to insufficient data on alcohol consumption status for pre-diagnostic plasma and their matched controls (**Table S4**), the impact of alcohol consumption on TRAF2 level could not be further assessed.

## Discussion

Blood samples are one of the most commonly used liquid biopsies. Within these samples promising biomarkers such as proteins, miRNAs, exosomes, metabolites, circulating tumor cells and circulating tumor DNA can be found (15). We have previously reported that circulating proteins are more promising than circulating miRNAs to detect SCCOT (8). In the present study, we expanded our investigations by measuring additional circulating proteins and including pre-diagnostic samples from individuals who subsequently developed SCCOT. A total of 261 unique proteins related to inflammation and/or cancer were evaluated in patient samples and matched controls without any history of cancer.

As indicated by PCA, protein profiles in plasma samples were similar between cases and controls. According to OPLS-DA, 21 proteins were identified as discriminant factors that separate diagnostic plasma from matched controls. However, only a handful of proteins were suggested as discriminatory in pre-diagnostic plasma. These pre-diagnostic samples had been collected within a broad time-span prior to the clinical diagnosis of SCCOT, and it was evident that the closer the time to diagnosis, the higher the degree of change in plasma proteins. TRAF2 was the only protein identified in both < 5 years pre-diagnostic and diagnostic samples. AUC values indicated the potential of TRAF2 in prediction and diagnosis of SCCOT. Notably, TRAF2 levels decreased in pre-diagnostic plasma, whereas they increased at diagnosis, indicating varying levels during cancer development.

Tumor necrosis factor (TNF) is a major inflammatory cytokine having both positive and negative effects on cancer (16). By activation of two receptors (TNFR1 which is ubiquitously expressed and TNFR2 which shows restricted expression) and subsequent recruitment of adaptor proteins, TNF can activate multiple signal transduction pathways involved in inflammation, cell survival or cell death, depending on the cellular context (16). TRAF2 can be recruited to both receptors and is a key player in dictating the outcome of TNF stimulation when both receptors are expressed on the same cell (17). Stimulation of TNFR1 and TNFR2 recruits TRAF2 and leads to canonical NF-κB and JNK kinase signaling to drive inflammation and cell survival. However, prolonged TNF stimulation of TNFR2 causes TRAF2 degradation and subsequent activation of the non-canonical NF-κB pathway. Degradation and release of TRAF2 from TNFR1 can in turn induce caspase-8 dependent apoptosis (17). It has been shown that TRAF2 is a frequently amplified oncogene in human epithelial cancers (18). With cancer development being a highly complex process and TRAF2 having varying roles under different cellular states, it is interesting to see the variation of TRAF2 levels in the plasma samples studied here.

It is worth noting that TRAF2 levels were significantly downregulated in patients with alcohol consumption compared to non-alcohol patients. Excessive alcohol consumption is a well-known risk factor for cancer development, including head and neck cancer (19). Prolonged alcohol exposure activates monocytes and macrophages, resulting in an increased production of pro-inflammatory cytokines such as TNF, which could contribute to tumor initiation and progression (20). The lower TRAF2 levels in patients with alcohol consumption could thus indicate degradation of TRAF2 in response to prolonged TNF stimulation. The potential role of alcohol consumption on TRAF2 levels can, however, not be clarified based on the present data.

To identify biomarkers for early detection of cancer, it is vital to perform longitudinal studies comprising asymptomatic individuals who are diagnosed with cancer at a later date. However, the majority of biomarker studies only analyze samples from patients at diagnosis compared to healthy controls. In a recent large scale retrospective longitudinal study, it was shown that cancer can be non-invasively detected up to four years before conventional diagnosis using a DNA methylation-based blood test (21). In another study using pre-diagnostic plasma samples from gastric cancer patients, increased HGF levels associated with cancer risk 6 years or longer prior to diagnosis (22). However, in our study, no increase in HGF levels were seen before diagnosis of SCCOT. Recently, using samples from the same biobank as in our study, Harlid et al. reported that it is challenging to develop effective biomarkers for early detection of colorectal cancer from plasma protein profiles in pre-diagnostic samples (23).

Ideally included more samples, especially pre-diagnostic plasma collected up to 5 years before diagnosis, should have been included in the analysis. Plasma samples collected from the same individual over time should also be investigated in order to adjust the effect of non-cancer-specific factors, such as aging and changes in lifestyles. Dynamic changes in protein levels and degree of variation should also be clarified.

In summary, we demonstrated that plasma protein profiles were altered in patients diagnosed with SCCOT and that only some of the alterations were detectable in circulation before a tumor was visible. Variation in protein levels during cancer development is important to map for the identification of predictive biomarkers. Furthermore, the potential impact of alcohol on TRAF2 levels needs further investigation.

## Data Availability Statement

The raw data supporting the conclusions of this article will be made available by the authors, without undue reservation.

## Ethics Statement

The studies involving human participants were reviewed and approved by Regional Ethics Committee, Sweden. The patients/participants provided their written informed consent to participate in this study.

## Author Contributions

Conceptualization, XG and KN. Data curation, XG. Formal analysis, XG and LW. Funding acquisition, KN and PC. Methodology, XG and KN. Project administration, KN. Supervision, KN. Writing - original draft, XG and KN. Writing - review & editing, XG, PC, BE, AS, NS, KZ, and KN. All authors contributed to the article and approved the submitted version.

## Funding

This study was supported by Lion’s Cancer Research Foundation, Umeå University; The Swedish Cancer Society (contract number 20 0754 PjF 01H); Umeå University; Region Västerbotten; Ministry of Health Czech Republic, conceptual development of research organization (MMCI, 00209805).

## Conflict of Interest

The authors declare that the research was conducted in the absence of any commercial or financial relationships that could be construed as a potential conflict of interest.

## Publisher’s Note

All claims expressed in this article are solely those of the authors and do not necessarily represent those of their affiliated organizations, or those of the publisher, the editors and the reviewers. Any product that may be evaluated in this article, or claim that may be made by its manufacturer, is not guaranteed or endorsed by the publisher.
