# Supplementary material for: Variation in Plasma Levels of TRAF2 Protein During Development of Squamous Cell Carcinoma of the Oral Tongue
Source: Front Oncol. 2021 Nov 23;11:753699. doi: 10.3389/fonc.2021.753699 (PMC8649619; doi:10.3389/fonc.2021.753699)
Supplement: Supplementary file 1 [file DataSheet_1.pdf]

Table S1. List of 276 assays from three Olink Multiplex panels.

CELL REGULATION (v.3702)			IMMUNE RESPONSE (v.3202)			IMMUNO-ONCOLOGY (v.3111)		
**Assay**	Uniprot ID	OlinkID	**Assay**	Uniprot ID	OlinkID	**Assay**	Uniprot ID	OlinkID
**AGR3**	Q8TD06	OID01369	**AREG**	P15514	OID01009	**ADA**	P00813	OID00775
**AMIGO2**	Q86SJ2	OID01365	**ARNT**	P27540	OID01000	**ADGRG1**	Q9Y653	OID00764
**APBB1IP**	Q7Z5R6	OID01364	**BACH1**	O14867	OID00996	**ANGPT1**	Q15389	OID00760
**ARHGAP1**	Q07960	OID01352	**BIRC2**	Q13490	OID00979	**ANGPT2**	O15123	OID00822
**ARHGEF12**	Q9NZN5	OID01390	**BTN3A2**	P78410	OID01027	**ARG1**	P05089	OID00815
**ARSB**	P15848	OID01337	**CCL11**	P51671	OID00970	**CAIX**	Q16790	OID00773
**ATG4A**	Q8WYN0	OID01370	**CD28**	P10747	OID00977	**CASP-8**	Q14790	OID00827
**BCL2L11**	O43521-2	OID01316	**CD83**	Q01151	OID01025	**CCL17**	Q92583	OID00821
**BCR**	P11274	OID01336	**CDSN**	Q15517	OID00960	**CCL19**	Q99731	OID00794
**BGN**	P21810	OID01339	**CKAP4**	Q07065	OID00985	**CCL20**	P78556	OID00837
**BOC**	Q9BWV1	OID01380	**CLEC4A**	Q9UMR7	OID00951	**CCL23**	P55773	OID00811
**CAMKK1**	Q8N5S9	OID01368	**CLEC4C**	Q8WTT0	OID00949	**CCL3**	P10147	OID00813
**CBL**	P22681	OID01340	**CLEC4D**	Q8WXI8	OID00988	**CCL4**	P13236	OID00796
**CDNF**	Q49AH0	OID01359	**CLEC4G**	Q6UXB4	OID00944	**CD244**	Q9BZW8	OID00758
**CFC1**	P0CG37	OID01335	**CLEC6A**	Q6EIG7	OID01017	**CD27**	P26842	OID00800
**CLSTN3**	Q9BQT9	OID01379	**CLEC7A**	Q9BXN2	OID01016	**CD28**	P10747	OID00793
**COL4A1**	P02462	OID01330	**CNTNAP2**	Q9UHC6	OID00943	**CD4**	P01730	OID00776
**CPXM1**	Q96SM3	OID01378	**CXADR**	P78310	OID00992	**CD40**	P25942	OID00781
**CRISP2**	P16562	OID01338	**CXCL12**	P48061	OID01008	**CD40-L**	P29965	OID00756
**CRX**	O43186	OID01315	**DAPP1**	Q9UN19	OID01011	**CD5**	P06127	OID00812
**DCBLD2**	Q96PD2	OID01376	**DCBLD2**	Q96PD2	OID01005	**CD70**	P32970	OID00808
**DCTN2**	Q13561	OID01356	**DCTN1**	Q14203	OID00958	**CD83**	Q01151	OID00841
**DDAH1**	O94760	OID01323	**DDX58**	O95786	OID01018	**CD8A**	P01732	OID00772
**DKKL1**	Q9UK85	OID01394	**DFFA**	O00273	OID01004	**CRTAM**	O95727	OID00766
**DNAJB1**	P25685	OID01342	**DGKZ**	Q13574	OID00948	**CSF-1**	P09603	OID00843
**ENTPD6**	O75354	OID01322	**DPP10**	Q8N608	OID00956	**CX3CL1**	P78423	OID00806
**FAM19A5**	Q7Z5A7	OID01363	**EDAR**	Q9UNE0	OID00946	**CXCL1**	P09341	OID00786
**FGF-21**	Q9NSA1	OID01387	**EGLN1**	Q9GZT9	OID00972	**CXCL10**	P02778	OID00807
**FLI1**	Q01543	OID01348	**EIF4G1**	Q04637	OID00976	**CXCL11**	O14625	OID00767
**GALNT2**	Q10471	OID01353	**EIF5A**	P63241	OID00975	**CXCL12**	P48061	OID00824
**GCG**	P01275	OID01329	**FAM3B**	P58499	OID01001	**CXCL13**	O43927	OID00830
**GCNT1**	Q02742	OID01349	**FCRL3**	Q96P31	OID00984	**CXCL5**	P42830	OID00801
**GFRA2**	O00451	OID01311	**FCRL6**	Q6DN72	OID01006	**CXCL9**	Q07325	OID00771
**GH2**	P01242	OID01328	**FGF2**	P09038	OID00954	**DCN**	P07585	OID00817
**GKN1**	Q9NS71	OID01386	**FXYD5**	Q96DB9	OID00962	**EGF**	P01133	OID00759
**GSAP**	A4D1B5	OID01308	**GALNT3**	Q14435	OID00961	**FASLG**	P48023	OID00792

**HS3ST3B1**	Q9Y662	OID01398	**GLB1**	P16278	OID00937	**FGF2**	P09038	OID00770
**HS6ST1**	O60243	OID01320	**HCLS1**	P14317	OID00942	**Gal-1**	P09382	OID00798
**IGSF3**	O75054	OID01321	**HEXIM1**	O94992	OID00987	**Gal-9**	O00182	OID00779
**IL17RB**	Q9NRM6	OID01385	**HNMT**	P50135	OID00969	**GZMA**	P12544	OID00804
**IQGAP2**	Q13576	OID01357	**HSD11B1**	P28845	OID00980	**GZMB**	P10144	OID00840
**JUN**	P05412	OID01332	**ICA1**	Q05084	OID01003	**GZMH**	P20718	OID00783
**KAZALD1**	Q96I82	OID01374	**IFNLR1**	Q8IU57	OID01010	**HGF**	P14210	OID00803
**KLK12**	Q9UKR0	OID01395	**IL10**	P22301	OID00993	**HO-1**	P09601	OID00805
**LGALS7**	P47929	OID01344	**IL12RB1**	P42701	OID01019	**ICOSLG**	O75144	OID00828
**LRMP**	Q12912	OID01354	**IL5**	P05113	OID01024	**IFN-gamma**	P01579	OID05552
**LRRN1**	Q6UXK5	OID01362	**IL6**	P05231	OID00947	**IL-1 alpha**	P01583	OID00757
**LYAR**	Q9NX58	OID01388	**IRAK1**	P51617	OID00950	**IL10**	P22301	OID00809
**LYPD1**	Q8N2G4	OID01367	**IRAK4**	Q9NWZ3	OID00940	**IL12**	P29459, P29460	OID00842
**MAP2K6**	P52564	OID01345	**IRF9**	Q00978	OID00945	**IL12RB1**	P42701	OID00835
**METAP1D**	Q6UB28	OID01360	**ITGA11**	Q9UKX5	OID01021	**IL13**	P35225	OID00836
**MOG**	Q16653	OID01358	**ITGA6**	P23229	OID00959	**IL15**	P40933	OID05551
**MRC2**	Q9UBG0	OID01392	**ITGB6**	P18564	OID01026	**IL18**	Q14116	OID00782
**NCLN**	Q969V3	OID01373	**ITM2A**	O43736	OID00968	**IL2**	P60568	OID00778
**NFATC1**	O95644	OID01325	**JUN**	P05412	OID00986	**IL33**	O95760	OID00788
**NFKBIE**	O00221	OID01310	**KLRD1**	Q13241	OID00995	**IL4**	P05112	OID00833
**NINJ1**	Q92982	OID01372	**KPNA1**	P52294	OID01022	**IL5**	P05113	OID00802
**OMG**	P23515	OID01341	**KRT19**	P08727	OID00967	**IL6**	P05231	OID00763
**OPTC**	Q9UBM4	OID01393	**LAG3**	P18627	OID01023	**IL7**	P13232	OID00761
**PAK4**	O96013	OID01327	**LAMP3**	Q9UQV4	OID01015	**IL8**	P10145	OID00752
**PCDH17**	O14917	OID01314	**LILRB4**	Q8NHJ6	OID00965	**KIR3DL1**	P43629	OID05550
**PFKM**	P08237	OID01333	**LY75**	O60449	OID00974	**KLRD1**	Q13241	OID00839
**PODXL2**	Q9NZ53	OID01389	**MASP1**	P48740	OID01014	**LAG3**	P18627	OID05553
**PRDX6**	P30041	OID01343	**MGMT**	P16455	OID00990	**LAMP3**	Q9UQV4	OID00826
**PREB**	Q9HCU5	OID01384	**MILR1**	Q7Z6M3	OID00971	**LAP TGF-beta-1**	P01137	OID00785
**PROK1**	P58294	OID01347	**NCR1**	O76036	OID01007	**MCP-1**	P13500	OID00765
**RGS8**	P57771	OID01346	**NF2**	P35240	OID00981	**MCP-2**	P80075	OID00795
**SEMA4C**	Q9C0C4	OID01381	**NFATC3**	Q12968	OID00973	**MCP-3**	P80098	OID00755
**SEZ6L2**	Q6UXD5	OID01361	**NTF4**	P34130	OID00966	**MCP-4**	Q99616	OID00768
**SIGLEC10**	Q96LC7	OID01375	**PADI2**	Q9Y2J8	OID01012	**MIC-A/B**	Q29983, Q29980	OID00820
**SIGLEC6**	O43699	OID01317	**PIK3AP1**	Q6ZUJ8	OID00997	**MMP12**	P39900	OID00829
**SKAP1**	Q86WV1	OID01366	**PLXNA4**	Q9HCM2	OID00982	**MMP7**	P09237	OID00814
**SLAMF8**	Q9P0V8	OID01391	**PPP1R9B**	Q96SB3	OID00936	**MUC-16**	Q8WXI7	OID05549
**SLITRK2**	Q9H156	OID01382	**PRDX1**	Q06830	OID00952	**NCR1**	O76036	OID00816

**SLITRK6**	Q9H5Y7	OID01383	**PRDX3**	P30048	OID00953	**NOS3**	P29474	OID00777
**SORCS2**	Q96PQ0	OID01377	**PRDX5**	P30044	OID00955	**PDCD1**	Q15116	OID00791
**STX16**	O14662	OID01312	**PRKCQ**	Q04759	OID00989	**PDGF subunit B**	P01127	OID00790
**STX6**	O43752	OID01318	**PSIP1**	O75475	OID00938	**PD-L1**	Q9NZQ7	OID00799
**SULT2A1**	Q06520	OID01351	**PTH1R**	Q03431	OID00978	**PD-L2**	Q9BQ51	OID00831
**TACC3**	Q9Y6A5	OID01399	**SH2B3**	Q9UQQ2	OID00983	**PGF**	P49763	OID00762
**TACSTD2**	P09758	OID01334	**SH2D1A**	O60880	OID01002	**PTN**	P21246	OID00823
**TCL1B**	O95988	OID01326	**SIT1**	Q9Y3P8	OID01013	**TIE2**	Q02763	OID00754
**TDRKH**	Q9Y2W6	OID01397	**SPRY2**	O43597	OID00998	**TNF**	P01375	OID05554
**TNFRSF10A**	O00220	OID01309	**SRPK2**	P78362	OID00994	**TNFRSF12A**	Q9NP84	OID00810
**TP53**	P04637	OID01331	**STC1**	P52823	OID00999	**TNFRSF21**	O75509	OID00818
**VAMP5**	O95183	OID01324	**TANK**	Q92844	OID01020	**TNFRSF4**	P43489	OID00819
**VEGFD**	O43915	OID01319	**TPSAB1**	Q15661	OID00941	**TNFRSF9**	Q07011	OID00753
**WASF1**	Q92558	OID01371	**TRAF2**	Q12933	OID00963	**TNFSF14**	O43557	OID00787
**WASF3**	Q9UPY6	OID01396	**TREM1**	Q9NP99	OID00991	**TRAIL**	P50591	OID00769
**WNT9A**	O14904	OID01313	**TRIM21**	P19474	OID00964	**TWEAK**	O43508	OID00789
**ZBTB16**	Q05516	OID01350	**TRIM5**	Q9C035	OID00957	**VEGFA**	P15692	OID00832
**ZBTB17**	Q13105	OID01355	**ZBTB16**	Q05516	OID00939	**VEGFR-2**	P35968	OID00780
