# Supplementary material for: Variation in Plasma Levels of TRAF2 Protein During Development of Squamous Cell Carcinoma of the Oral Tongue
Source: Front Oncol. 2021 Nov 23;11:753699. doi: 10.3389/fonc.2021.753699 (PMC8649619; doi:10.3389/fonc.2021.753699)
Supplement: Supplementary file 2 [file DataSheet_2.pdf]

Table S2. Information on patient-control matchings.

Case set ID	Patient ID	Control ID	Match in sex	Match in age (± 5 years)	Match in sampling date (± 5 years)
1	43	C82	Yes	Yes	No
2	44	C83	Yes	Yes	No
3	45	C84	Yes	Yes	Yes
4	46	C85	Yes	Yes	Yes
5	47	C86	Yes	Yes	Yes
6	48	C87	Yes	Yes	Yes
7	49	C88	Yes	Yes	Yes
8	50	C89	Yes	Yes	Yes
9	51	C90	No	No	Yes
10	52	C91	Yes	Yes	Yes
11	53	C92	Yes	Yes	Yes
12	54	C93	Yes	No	Yes
13	55	C94	Yes	Yes	Yes
14	56	C95	Yes	No	Yes
15	57	C96	No	No	Yes
16	58	C97	Yes	Yes	Yes
17	59	C98	Yes	Yes	Yes
18	60	C99	Yes	Yes	No
19	61	C100	Yes	Yes	Yes
20	62	C101	Yes	Yes	Yes
21	63	C102	Yes	Yes	Yes
22	64	C103	Yes	Yes	Yes
23	65	C104	Yes	Yes	No
24	66	C105	Yes	Yes	Yes
25	67	C106	Yes	No	Yes
26	68	C107	Yes	Yes	Yes
27	69	C108	Yes	Yes	Yes
28	70	C109	Yes	Yes	No
