# Supplementary material for: Variation in Plasma Levels of TRAF2 Protein During Development of Squamous Cell Carcinoma of the Oral Tongue
Source: Front Oncol. 2021 Nov 23;11:753699. doi: 10.3389/fonc.2021.753699 (PMC8649619; doi:10.3389/fonc.2021.753699)
Supplement: Supplementary file 3 [file DataSheet_3.pdf]

Table S3. Performance of potential diagnostic biomarkers.

Protein	AUC	Std. Error	*p*-value	95% Confidence Interval		Cut-off level (NPX)	Sensitivity	Specificity
				Lower Bound	Upper Bound			
MMP7	0.82	0.055	0.000	0.71	0.93	9.87	0.75	0.71
TWEAK	0.81	0.062	0.000	0.69	0.93	8.29	0.75	0.79
LRRN1	0.81	0.056	0.000	0.70	0.92	4.40	0.75	0.71
CPXM1	0.79	0.062	0.000	0.67	0.91	4.40	0.71	0.75
ITGA11	0.77	0.062	0.001	0.64	0.89	2.26	0.79	0.57
HGF	0.76	0.064	0.001	0.64	0.89	8.28	0.71	0.71
ANGPT1	0.76	0.066	0.001	0.63	0.89	8.77	0.75	0.71
CLEC4D	0.74	0.068	0.002	0.61	0.87	2.44	0.64	0.68
ICOSLG	0.73	0.067	0.003	0.60	0.87	5.19	0.75	0.64
IL6	0.73	0.069	0.003	0.59	0.86	2.69	0.71	0.64
TRAF2	0.72	0.070	0.004	0.59	0.86	3.21	0.79	0.71
TNFSF14	0.72	0.068	0.004	0.59	0.86	3.62	0.68	0.64
FASLG	0.71	0.071	0.006	0.57	0.85	5.65	0.71	0.75
ATG4A	0.70	0.070	0.009	0.57	0.84	3.72	0.68	0.68
MCP-3	0.70	0.072	0.009	0.56	0.85	1.25	0.75	0.68
