# Supplementary material for: Variation in Plasma Levels of TRAF2 Protein During Development of Squamous Cell Carcinoma of the Oral Tongue
Source: Front Oncol. 2021 Nov 23;11:753699. doi: 10.3389/fonc.2021.753699 (PMC8649619; doi:10.3389/fonc.2021.753699)
Supplement: Supplementary file 4 [file DataSheet_4.pdf]

**Table S4**. Availability of alcohol consumption data

| | No alcohol | | Alcohol | | Unknown | | Total |
|---|---|---|---|---|---|---|---|
| | **Count** | **%** | **Count** | **%** | **Count** | **%** | **Count** |
| **Patients at diagnosis** | 6 | 21.40% | 19 | 67.90% | 3 | 10.70% | 28 |
| **Controls for patients at diagnosis** | 7 | 25.00% | 17 | 60.70% | 4 | 14.30% | 28 |
| **< 5 years pre-diagnosis** | 0 | 0.00% | 7 | 46.70% | 8 | 53.30% | 15 |
| **Controls for < 5 years pre-diagnosis** | 1 | 3.60% | 15 | 53.60% | 12 | 42.90% | 28 |
| **5-15 years pre-diagnosis** | 0 | 0.00% | 3 | 18.80% | 13 | 81.30% | 16 |
| **Controls for 5-15 years pre-diagnosis** | 1 | 3.10% | 5 | 15.60% | 26 | 81.30% | 32 |
| **>15 years pre-diagnosis** | 0 | 0.00% | 0 | 0.00% | 11 | 100.00% | 11 |
| **Controls for >15 years pre-diagnosis** | 0 | 0.00% | 0 | 0.00% | 21 | 100.00% | 21 |
