# Supplementary material for: Variation in Plasma Levels of TRAF2 Protein During Development of Squamous Cell Carcinoma of the Oral Tongue
Source: Front Oncol. 2021 Nov 23;11:753699. doi: 10.3389/fonc.2021.753699 (PMC8649619; doi:10.3389/fonc.2021.753699)
Supplement: Supplementary file 5 [file DataSheet_5.pdf]

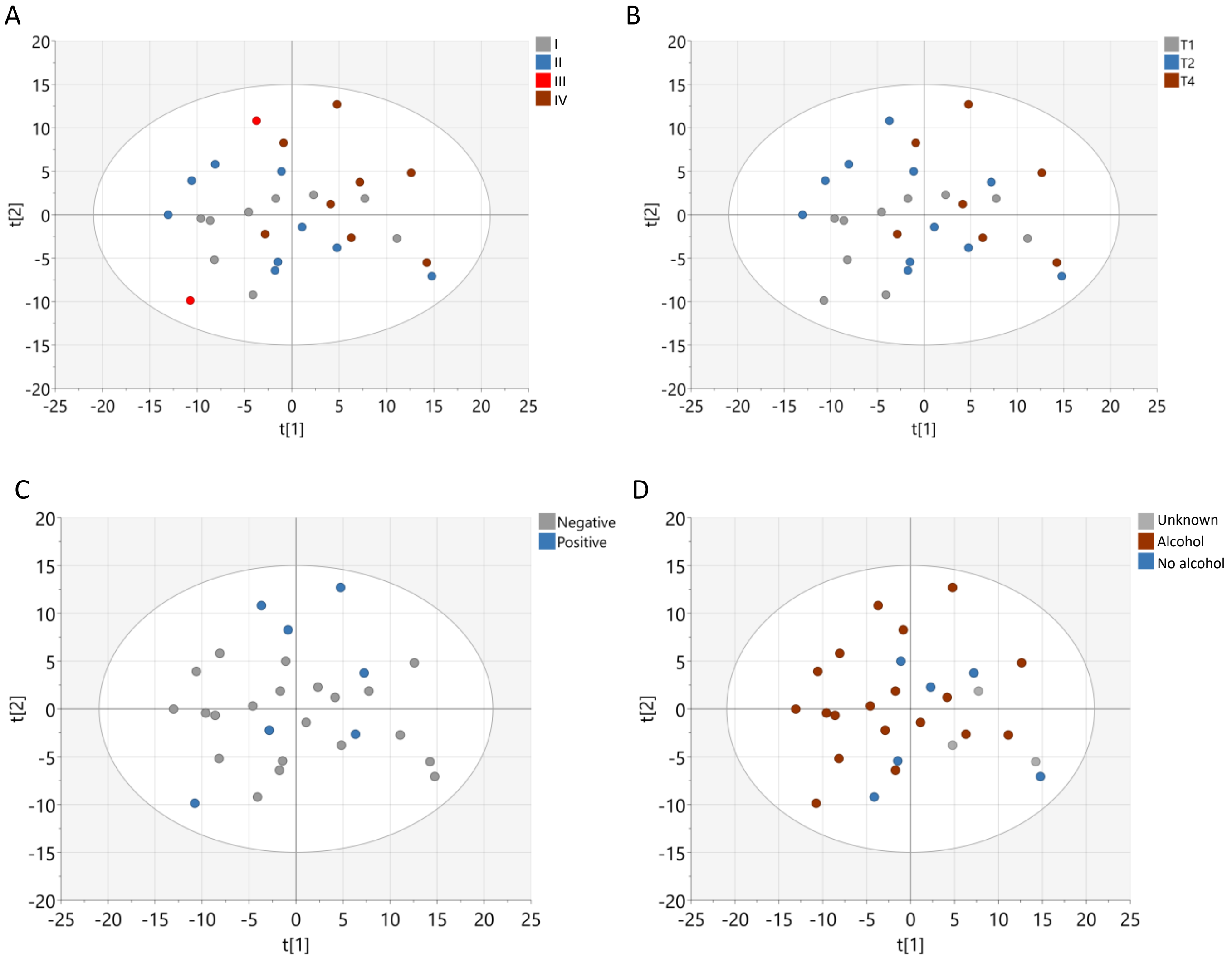

Figure S1. PCA plots visualizing protein expression profiles in plasma samples at diagnosis (Two components, R^2= 0.363, Q^2= 0.227). Samples are colored according to TNM stage (A), T stage (B), node status (C) and alcohol consumption status (D).
