# Supplementary material for: Variation in Plasma Levels of TRAF2 Protein During Development of Squamous Cell Carcinoma of the Oral Tongue
Source: Front Oncol. 2021 Nov 23;11:753699. doi: 10.3389/fonc.2021.753699 (PMC8649619; doi:10.3389/fonc.2021.753699)
Supplement: Supplementary file 6 [file DataSheet_6.pdf]

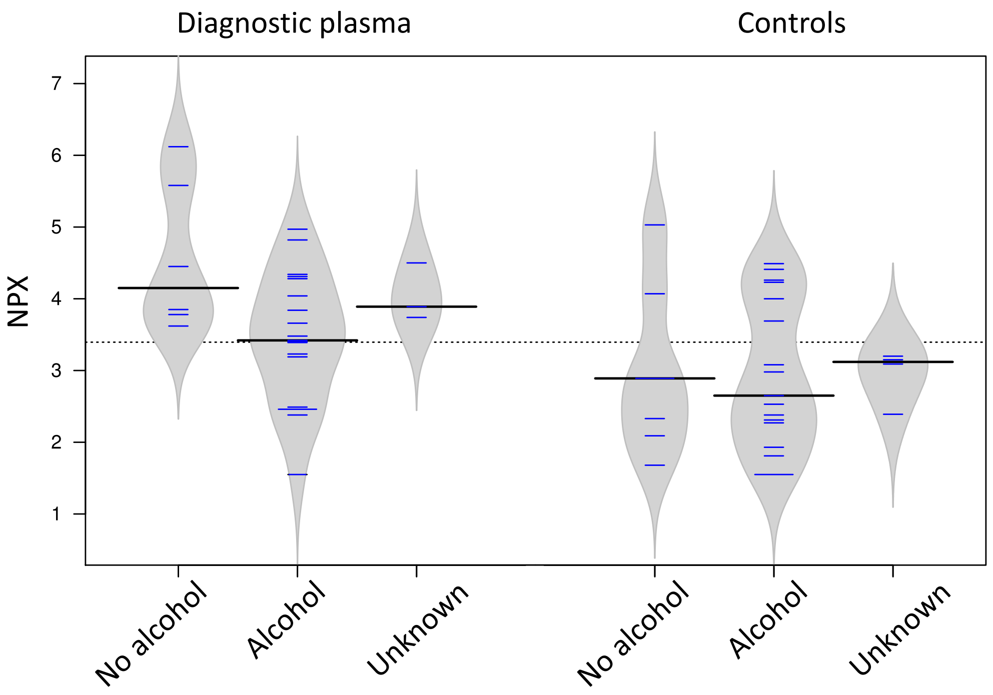

Figure S2. Bean plots showing TRAF2 protein levels in diagnostic plasma and the matched controls. Samples are sub-grouped according to status of alcohol consumption.
